# Global and regional drivers for exceptional climate extremes in 2023-2024: beyond the new normal

**DOI:** 10.1038/s41612-025-00996-z

**Published:** 2025-04-07

**Authors:** Shoshiro Minobe, Erik Behrens, Kirsten L. Findell, Norman G. Loeb, Benoit Meyssignac, Rowan Sutton

**Affiliations:** 1 Faculty of Science, Hokkaido University, Sapporo, Japan; 2 The National Institute of Water and Atmospheric Research, Wellington, New Zealand; 3 Geophysical Fluid Dynamics Laboratory, National Oceanic and Atmospheric Administration, Princeton, NJ USA; 4 NASA Langley Research Center, Hampton, VA USA; 5 Université de Toulouse, LEGOS (CNES/CNRS/IRD/UT3), Toulouse, France; 6 University of Reading and National Centre for Atmospheric Science, Reading, UK

**Keywords:** Climate change, Atmospheric science, Ocean sciences

## Abstract

Climate records have been broken with alarming regularity in recent years, but the events of 2023–2024 were exceptional even when accounting for recent climatic trends. Here we quantify these events across multiple variables and show how excess energy accumulation in the Earth system drove the exceptional conditions. Key factors were the positive decadal trend in Earth’s Energy Imbalance (EEI), persistent La Niña conditions beginning in 2020, and the switch to El Niño in 2023. Between 2022 and 2023, the heating from EEI was over 75% larger than during the onset of similar recent El Niño events. We show further how regional processes shaped distinct patterns of record-breaking sea surface temperatures in individual ocean basins. If the recent trend in EEI is maintained, we argue that natural fluctuations such as ENSO cycles will increasingly lead to amplified, record-breaking impacts, with 2023–2024 serving as a glimpse of future climate extremes.

## Introduction

As climate change advances, each year brings numerous broken climate records and uncharted climatic conditions^1–7^, engendering the sense that climatological norms are no longer representative of “normal”^8^. However, the conditions of 2023 and early 2024 stand out as extraordinary, even in the context of a new normal. Unprecedented summertime heat across the Northern Hemisphere brought catastrophic impacts to many regions of the globe, including heat waves, droughts, wildfires, and extreme rainfall and flooding^9–16^. The Paris Agreement established the objective to pursue efforts to limit global mean temperature increase to 1.5 °C above pre-industrial levels, but in 2023, more than two-thirds of individual days surpassed this target (https://climate.copernicus.eu/record-warm-november-consolidates-2023-warmest-year) and in 2024 annual mean air-temperature has exceeded this threshold for the first time (https://climate.copernicus.eu/copernicus-2024-first-year-exceed-15degc-above-pre-industrial-level). The ocean bore particularly dramatic signatures of extreme temperatures, with between 30% and 40% of the global ocean area experiencing a marine heat wave each month from April through December 2023^17–19^, and drastic decline of global sea-ice^20^. Here we show that the climate conditions of 2023 and early 2024 were exceptional even when recent climatic trends and large-scale climate variability are taken into account.

Whilst many timely publications provide important information about the anomalous conditions in 2023^19,21–28^, further efforts are needed to understand these exceptional climate conditions, their implications, and the potential for recurrence. We contribute to this effort in three novel ways. First, we propose and apply an objective statistical analysis method to determine significance of the recent extreme conditions while accounting for recent climatic trends and past variability. The “Abnormal record-Breaking (AB) test” (“Methods”, Supplementary Fig. 1) provides a robust, simple, and versatile statistical test which can be widely applied to climate variables and indicators to evaluate extreme conditions. Next, we quantify the contribution of the Earth’s energy imbalance (EEI) to the exceptional heat extremes observed in the ocean and atmosphere in 2023–2024 by comparing it to the onset of other major recent El Niño events. Our results show that the EEI contribution to the warming of the upper ocean and atmosphere exceeded previous events by 75%. Third, we provide further insight into two specific regions, the subtropical Northeastern Atlantic and the Southern Ocean, which show extreme conditions in 2023 linked to shortwave radiation and atmospheric circulation, respectively. We discuss the possible role of internal variability related to these events and highlight the need for further research on attribution of such extremes.

## Results

### Exceptional climate conditions

**Fig. 1:**
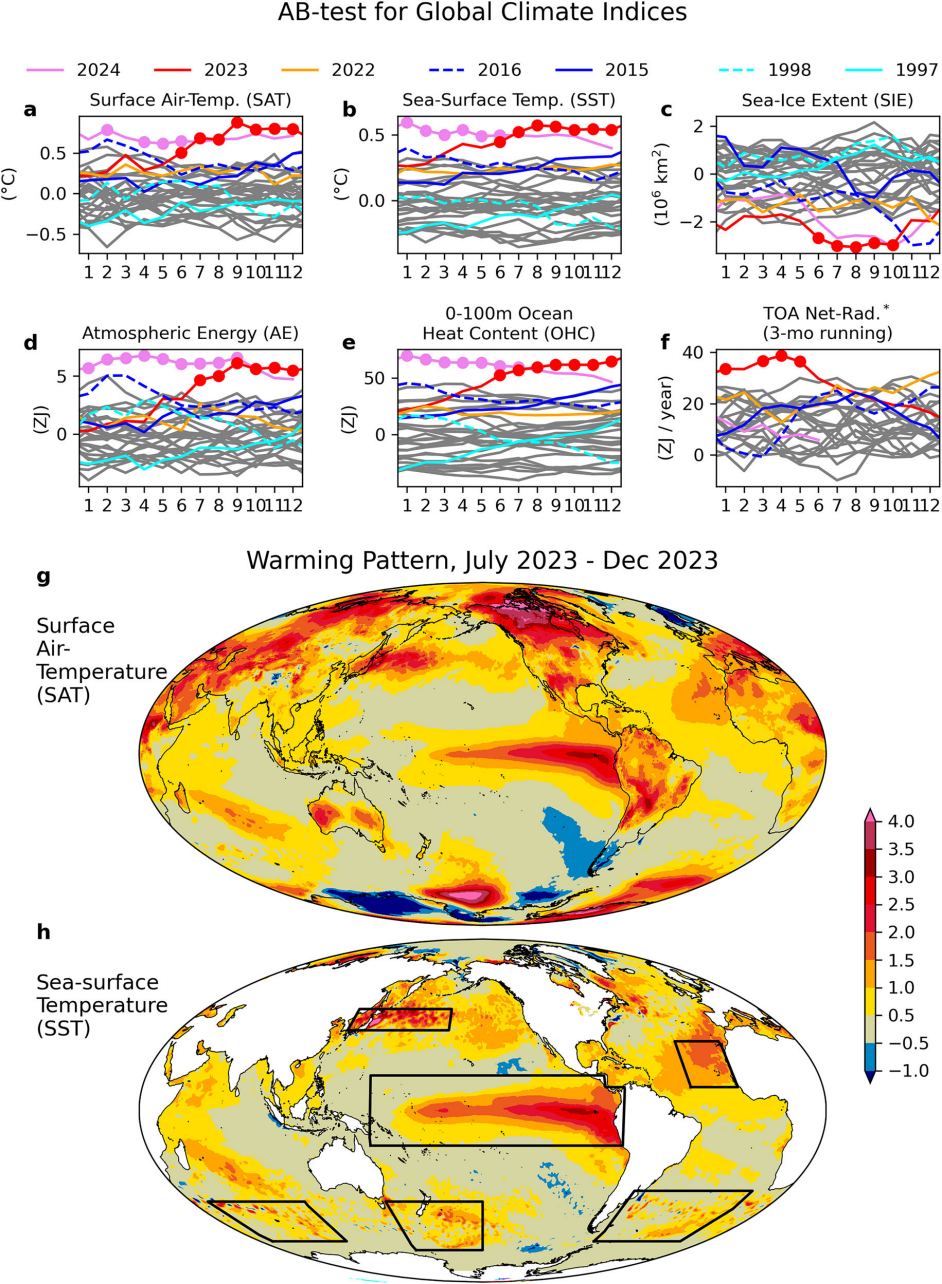
Major climate indices with AB-test results and global SAT/SST anomaly patterns from July to December 2023. Monthly values of global climate indices of **a** SAT, **b** SST, **c** SIE, **d** AE, **e** OHC in upper 100 m, and **f** TOA Net Radiation. Months that pass the AB-test are indicated by filled circles. The data shown are global averages for (**a**) and (**b**), and global integrals for (**c**)–(**f**). All fields are anomalies relative to the 1993–2022 climatology, except for TOA net radiation in (**f**), which is shown as a mean-retained anomaly (see “Methods”) to indicate the sign’s importance as an indicator of energy accumulation or loss, with a reference period of 2001–2022. The start year of the plot is 1993, except for TOA net-radiation which starts in March 2000. Previous super El Niño years (1997/1998 and 2015/2016) and recent 3 years (2022, 2023, and 2024) are shown by colored lines as indicated by the legend. July–December averaged **g** SAT and **h** SST anomalies over the globe.

Abnormal record-breaking conditions began in June of 2023 for two of the most widely used global climate indices: globally averaged surface air-temperature (SAT) (Fig. 1a) and sea-surface temperature (SST) (Fig. 1b). Global sea-ice extent (SIE) also exhibited abnormal record-breaking in mid-2023, mainly due to a reduction of sea ice around Antarctica (Fig. 1c). These results emphasize that the global climate in 2023 not only broke records, but also broke records by wide margins—even when accounting for the recent progression of global warming. Similar results to those for SAT and SST are found for atmospheric energy (AE) (Fig. 1d) and near-surface (0–100 m) ocean heat content (OHC) (Fig. 1e). These four variables (SAT, SST, AE, and OHC) are highly correlated (correlation coefficients between any two are above 0.85 when considering a 3-month running average), however, near-surface OHC anomalies are ten times larger than typical AE anomalies. This motivates our detailed OHC analyses in the subsequent sections as even small OHC changes have large impacts on AE and SAT/SST and how they evolve with time (Supplementary Fig. 2). Net top-of-atmosphere (TOA) radiation observations from the Clouds and the Earth’s Radiant Energy System (CERES) also exhibited abnormal record-breaking conditions in early 2023 (Fig. 1f). In the next section, we show that this exceptional heat was predominantly stored in the top 100 m of the ocean, which led to rapid increase in top-100 m OHC during this period.

**Fig. 2:**
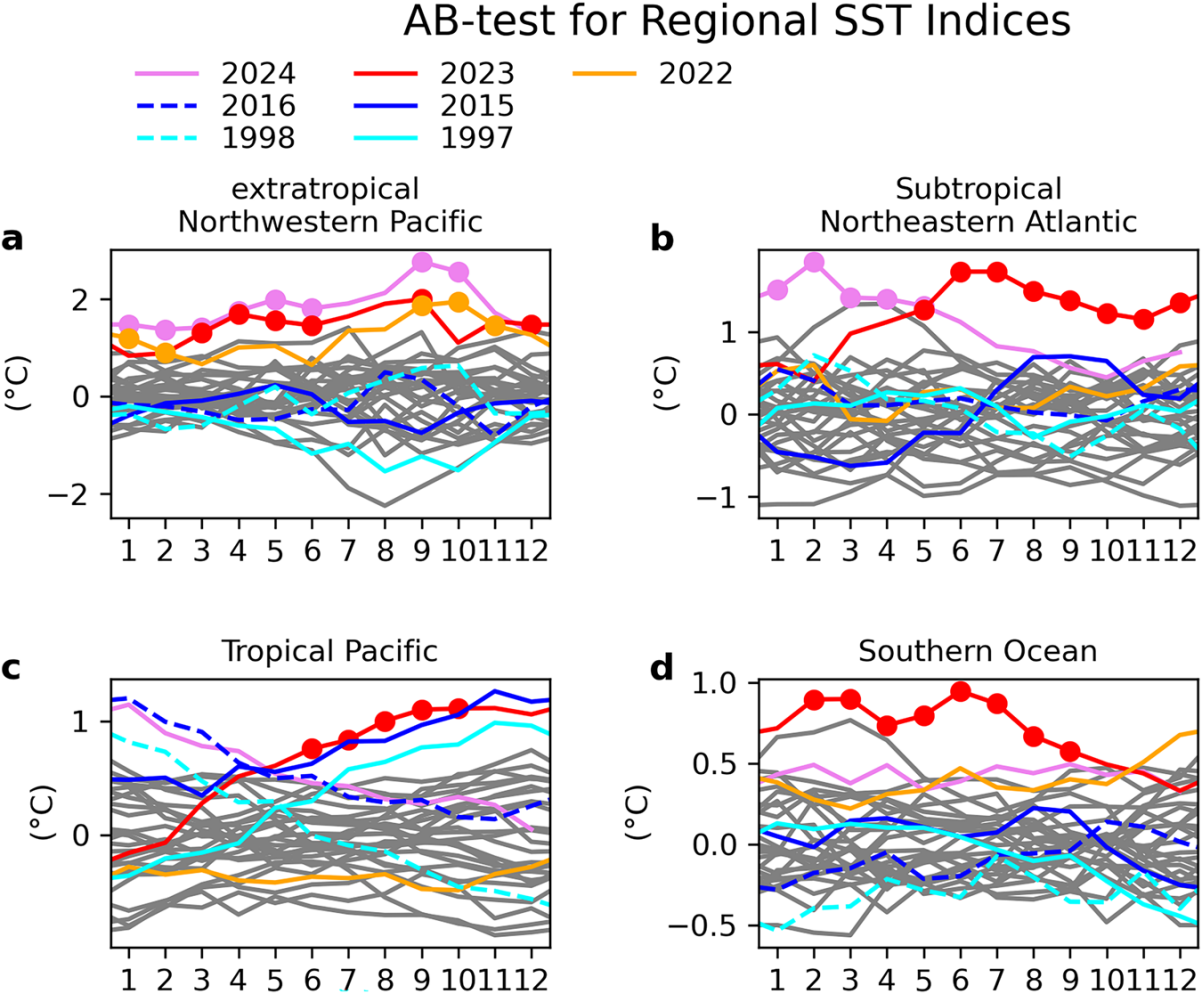
Monthly anomaly time series of SSTs with AB-test results in selected regions. The time series represent the extratropical Northwestern Pacific (**a**), the subtropical Northeastern Atlantic (**b**), the tropical Pacific (**c**), and the combined three-areas in the Southern Ocean (**d**) relative to the 1993 to 2022 climatology. The respective regions are shown by the boxes in Fig. 1h.

Temperature anomalies during the latter half of 2023 (July–December) show a distinctive spatial structure (Fig. 1g, h) that is quite different from the much more spatially uniform pattern of warming over the last ~75 years (Supplementary Fig. 3) and resembles a positive El Niño-Southern Oscillation (ENSO) phase in the tropics^29^. Regional SSTs averaged over each of the four regions in Fig. 1h (indicated by boxes) highlight different times of emergence of abnormal record-breaking conditions for each region (Fig. 2a–d).

SSTs in the extratropical Northwestern Pacific first exhibited abnormal record-breaking conditions in early 2022, with most months since September 2022 continuing through June 2024 passing the AB test. The subtropical Northeastern Atlantic first showed abnormal record-breaking conditions in May 2023, continuing unabated through May 2024. In the tropical Pacific, on the other hand, abnormal record-breaking conditions occurred between June and October 2023, though the anomalous SSTs in this region were on par with those observed during the 2015/16 Super El Niño (dark blue lines). However, neither of the two most common El Niño indices (i.e., Niño 3.4 and Niño 3) were record breaking in 2023 (Supplementary Fig. 4). The 2023 warming in the tropical Pacific is broader in latitude than the warming in the previous Super El Niños. Abnormal record-breaking conditions began in the Southern Ocean in February 2023, lasting through September 2023. The Southern Ocean anomalies reach abnormal record-breaking conditions first, at the start of the Southern hemisphere summer. The earlier emergence of AE anomalies in the Southern hemisphere is evident in Supplementary Fig. 2.

### Global energy perspective

**Fig. 3:**
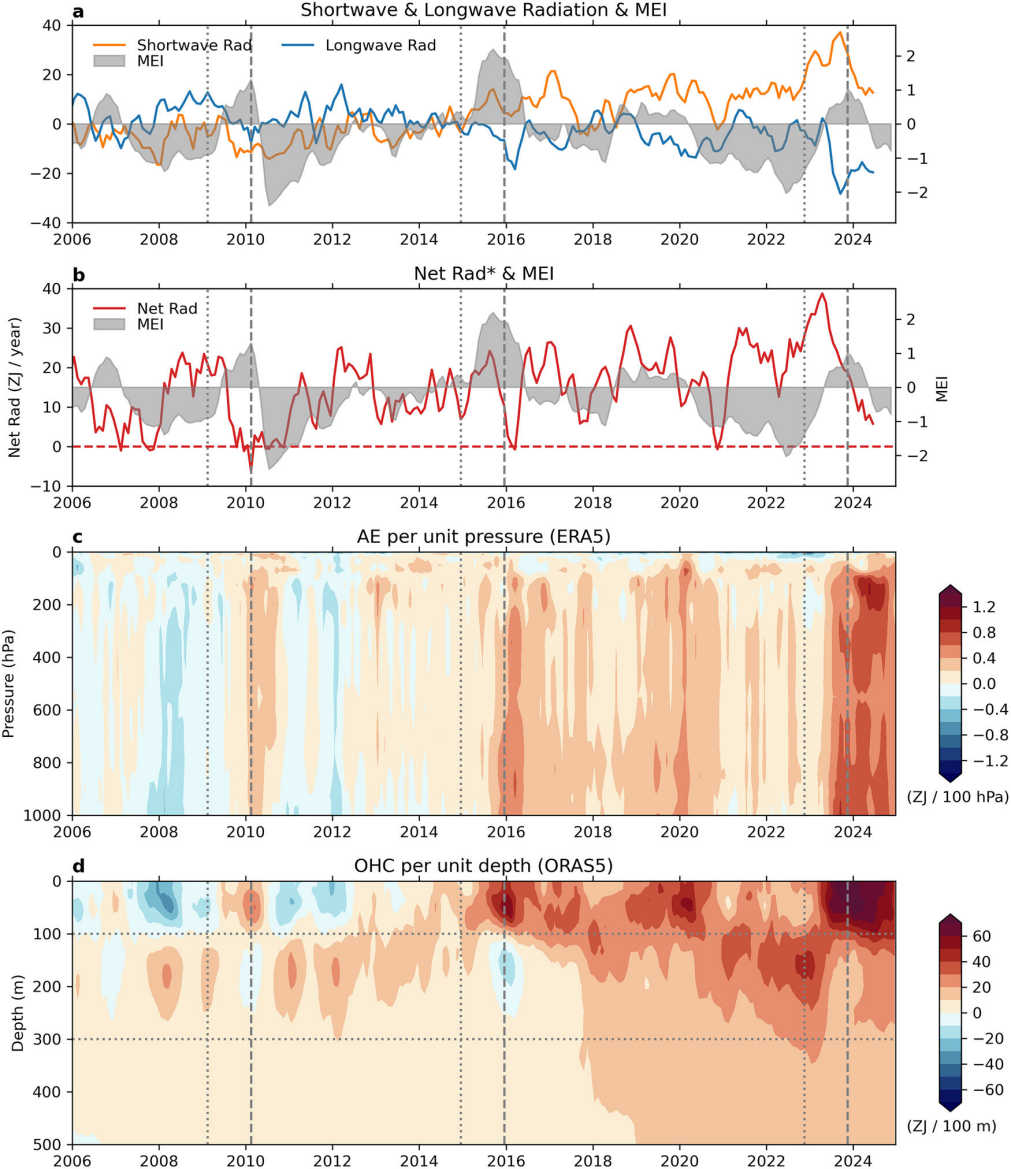
Global TOA radiation and heat content evolution. **a** Anomalies of downward shortwave and downward longwave radiation and multivariate ENSO Index (MEI); **b** mean-retained anomalies of TOA downward net radiation or EEI and MEI; **c** AE anomalies per unit pressure; and **d** OHC anomalies per unit depth for the Argo period since 2006. Anomalies in CERES data are calculated relative to 2001–2022, while anomalies in other data are calculated relative to 1993–2022. Time series shown in **a** and **b** are smoothed by a 3-month running average. The vertical gray dashed and dotted lines in each panel indicate the 1-year period over which the heat budget analysis in Fig. 4 is conducted for each of three El Niño events.

What led to the record-breaking warmth of 2023? A central factor is Earth’s energy budget, which describes the difference between incoming solar radiant energy absorbed by Earth and outgoing thermal infrared radiation emitted to space (Fig. 3a, b). Both quantities show large fluctuations on interannual times scales associated with ENSO fluctuations, consistent with earlier literature^30–32^. During El Niño phases the EEI drops rapidly, even turning negative during the 2010 and 2016 events, indicating a net Earth’s energy loss. However, over the past two decades, an exceptional trend in EEI (Fig. 3b) has been observed from satellite TOA radiation, in-situ ocean, and satellite altimetry and space gravimetry measurements^3,4,33,34^. This extra energy input has rendered the system significantly warmer, particularly within the ocean below 100 m (see Fig. 3d). This prolonged build-up of energy into the climate system is associated with an unprecedented increase in TOA absorbed solar radiation (ASR) that is only partially compensated by a weaker increase in outgoing longwave radiation (Fig. 3a). The ASR changes have been linked to decreases in low and middle cloud fraction in middle-to-high latitudes in the northern hemisphere and decreases in middle cloud fraction in the southern hemisphere^1^.

From mid-2020 to mid-2023, three consecutive years of La Niña conditions contributed to a further increase in TOA net downward radiation, injecting about 68 ZJ of energy into the system, equivalent to 23% of the total energy accumulation for 2000–2023 (Fig. 3a, b). During the first part of 2023, the net TOA flux set a record-breaking abnormal increase between December 2022 and June 2023 (Fig. 1f), followed by a decrease at the onset of the El Niño in May 2023. Near-surface OHC (top 100 m layer) within the ocean and AE also increased between December 2022 and June 2023, and then intensified further during the 2023 El Niño event (Fig. 1d, e).

These changes align with the expected energetic impacts associated with the growth and decay phases of El Niño in the tropics^35–40^. Specifically, as is common during the transition from La Niña to El Niño conditions, early 2023 is marked by positive SST anomalies in the Eastern and Central Pacific, coinciding with a deepening of the thermocline in the eastern and central Pacific and a shallowing of the thermocline in the western Pacific, likely driven by wind forcing. This flattening of the thermocline (Fig. 3d) leads mechanically to an increase in the near-surface OHC (0–100 m) and a decrease between 100 m and 300 m (Supplementary Fig. 5) (see also refs. ^38,41^). Changes in AE follow those in the 0–100 m OHC layer a few months later (Fig. 3c and Supplementary Fig. 5).

**Fig. 4:**
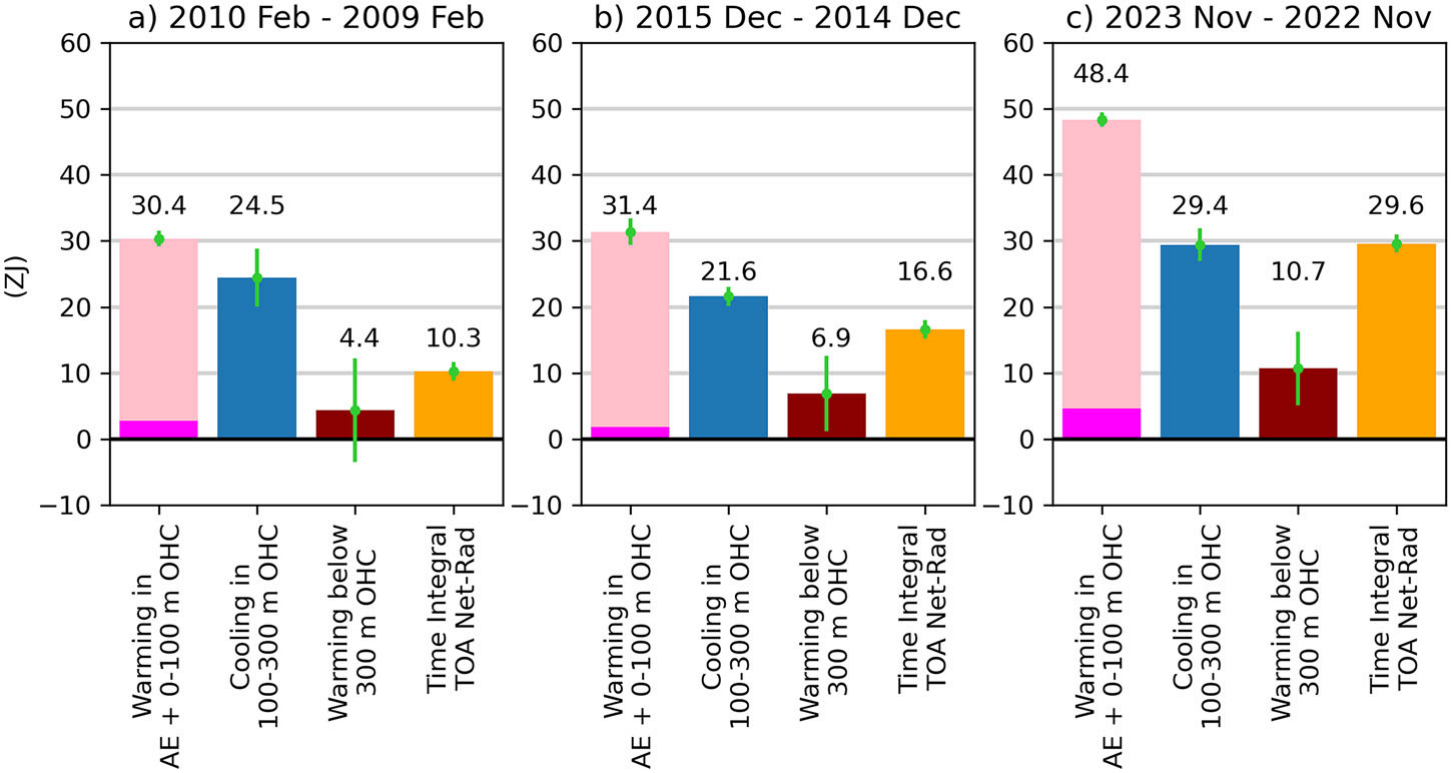
Warming in AE (magenta) and OHC in the near-surface (0–100 m; pink) and below 300 m (dark red), cooling in subsurface (100–300 m) OHC (blue), and time-integrated TOA net radiation (orange) for 1-year periods. These periods —**a** 2010 Feb–2009 Feb, **b** 2015 Dec–2014 Dec, and **c** 2023 Nov–2022 Nov— are selected to capture the strongest 1-year warming in 0–100 m OHC associated with each El Niño event. The OHC changes are calculated from the difference between two 3-month averages separated by 1 year (e.g., the difference in **a** is between the mean of Jan–Feb–March 2009 and that of 2010), while the TOA net radiation is integrated over the 1-year period between the midpoints of these 3-month intervals. Uncertainty (see “Methods”) of each respective estimate is indicated by green lines. The number above each bar indicates the height of the bar in units of ZJ.

While the vertical redistribution of heat within the ocean during the 2023 El Niño is similar to that observed during the 2010 and 2016 major El Niño events, heating of the near-surface layer is markedly different (Fig. 4 and Supplementary Table 2). Our selection of the previous ENSO events is limited by the observed OHC record through Argo, which became operational in 2006^42^ (Supplementary Fig. 6) The cooling between 100 m and 300 m depths and the integration of TOA net radiation are of similar magnitudes for the recent warming event. However, the increase in EEI between the current event and previous events is significantly larger than the increase in 100–300 m cooling. Specifically, the cooling between 100–300 m from November 2022 to November 2023 surpasses the previous maximum cooling (February 2009 to February 2010) by only 4.8 ZJ. In contrast, TOA net radiation from November 2022 to November 2023 is 13 ZJ higher than the previous peak heating (December 2014 to December 2015), marking an increase of over 75%. This extra heat is largely stored in the atmosphere and the upper ocean, as evidenced by the observation that the increases in combined AE and 0–100 m OHC were over 50% larger in the 2023 El Nino than occurred during the 2010 and 2016 El Niños.

The abnormal record-breaking conditions in 2023 thus resulted from the combination of the long-term positive EEI trend, the 3 year La Nina conditions, and the switch to El Niño. A recent analysis of outputs from the Climate Model Intercomparison Project Phase Six suggests that the transition from a multi-year La Nina to an El Nino substantially increase the EEI^43^. The long-term trend in EEI is due to a positive radiative forcing resulting from continued emissions of well-mixed greenhouse gases and reductions in aerosol emissions in some parts of the northern hemisphere due to air quality legislation^21,44–46^. A recent assessment suggests that climate models fail to capture the exceptional global mean temperature increase in 2023^47^ or the modeled probability is extremely low^40^. A key reason may lie in the models’ representation of the unprecedented observed changes in Earth’s energy budget. Clearly, further analysis is required to fully test the models.

### Regional extreme events

**Fig. 5:**
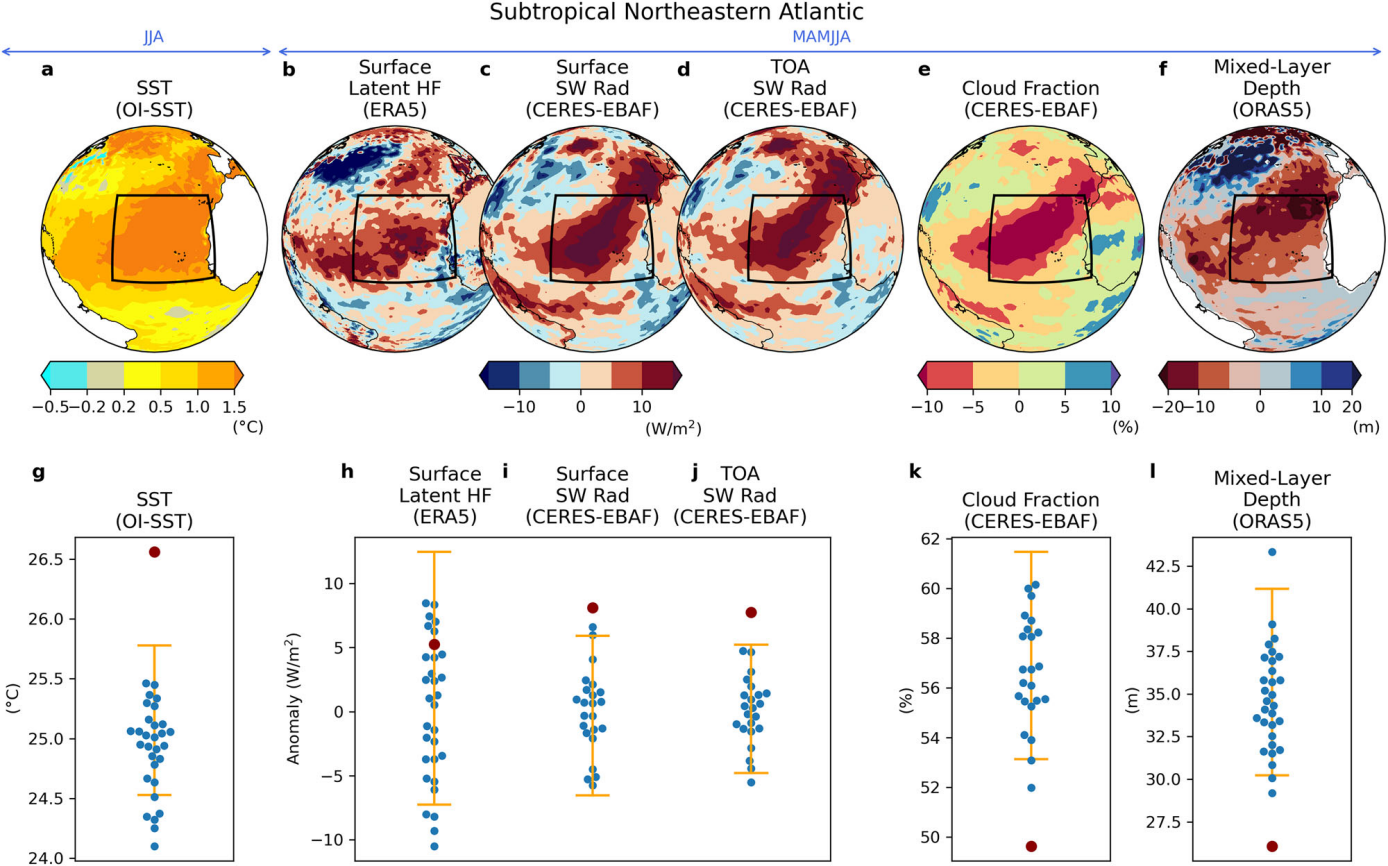
Subtropical Northeastern Atlantic anomalies in 2023 and interannual variability. (Top panels) Anomalies in the subtropical Northeastern Atlantic for **a** SST, **b** surface latent heat flux, **c** surface short-wave radiation, **d** top-of-atmosphere (TOA) shortwave radiation, **e** cloud fraction, and **f** mixed-layer depth. (Bottom panels) The corresponding area-averaged and seasonally-averaged data for each year (**g**) SST, (**h**) surface latent heat flux, (**i**) surface short-wave radiation, (**j**) TOA shortwave radiation, (**k**) cloud fraction, and (**l**) mixed-layer depth. In (**g**–**l**), the red dot represents the 2023 value, blue dots indicate 1993–2022 values, and yellow bars show the 5th–95th percentile of 2023 value estimation based on a linear regression model for the learning period between 1993 and 2022 for SST and mixed layer depth (**g**, **l**) and between 2000 and 2022 for CERES-EBAF data (**i**–**k**) due to the limited data availability and for latent HF (**h**) for consistency. The average range is between 10°–30°N and 40°–15°W.

In addition to its key role in the global heat budget, there is evidence in the CERES data that the exceptional TOA net radiation played an important role in regional SST anomalies over the subtropical Northeastern Atlantic (Fig. 5a) during boreal spring and summer 2023. When we examine shortwave and longwave radiation of CERES and latent and sensible heat fluxes from ERA5, the strongest heating is given by surface shortwave radiation, which is abnormal record-breaking and larger than the latent heat flux (Fig. 5b, c, h, i). The surface shortwave-radiation anomaly is consistent with the TOA shortwave radiation (Fig. 5d, j), and is accompanied by substantial reduction in cloud fraction (Fig. 5e, k), suggesting weakened cloud reflection resulted in increased shortwave radiation reaching the ocean.

Further analyses suggest that the reduced cloud fraction was mainly due to a decrease in low cloud (Supplementary Fig. 7). The important role of low cloud reduction in EEI increase is also emphasized by a recent study, which analyzed low cloud of ERA5 data^27^, whereas our results indicates that the low cloud reduction is also evident in MODIS data in this region (Supplementary Fig. 7). Aerosol optical thickness also exhibited a decrease in the south of the analysis area, but the pattern does not overlap well with the increase of the TOA shortwave radiation (Supplementary Fig. 7). In addition, the mixed layer depth was unusually shallow, displaying an abnormal record-breaking condition (Fig. 5f, i), likely the result of anomalously low winds in this region^24^ (Supplementary Fig. 7). This means that the surface temperature increases per unit heat flux (i.e. efficiency of the warming) was high in 2023. Although the ERA5 reanalysis data has some caveats^48–55^ in the analyzed fields, the results show consistency between them and point to the combined effect of an exceptionally weak wind and high surface shortwave radiation, in association with shallow mixed layer, as key factors for the temperature extremes over the subtropical Northeastern Atlantic. We note that there were also concurrent anomalies in atmospheric circulation (Supplementary Fig. 7), which would have contributed to the low wind speed.

**Fig. 6:**
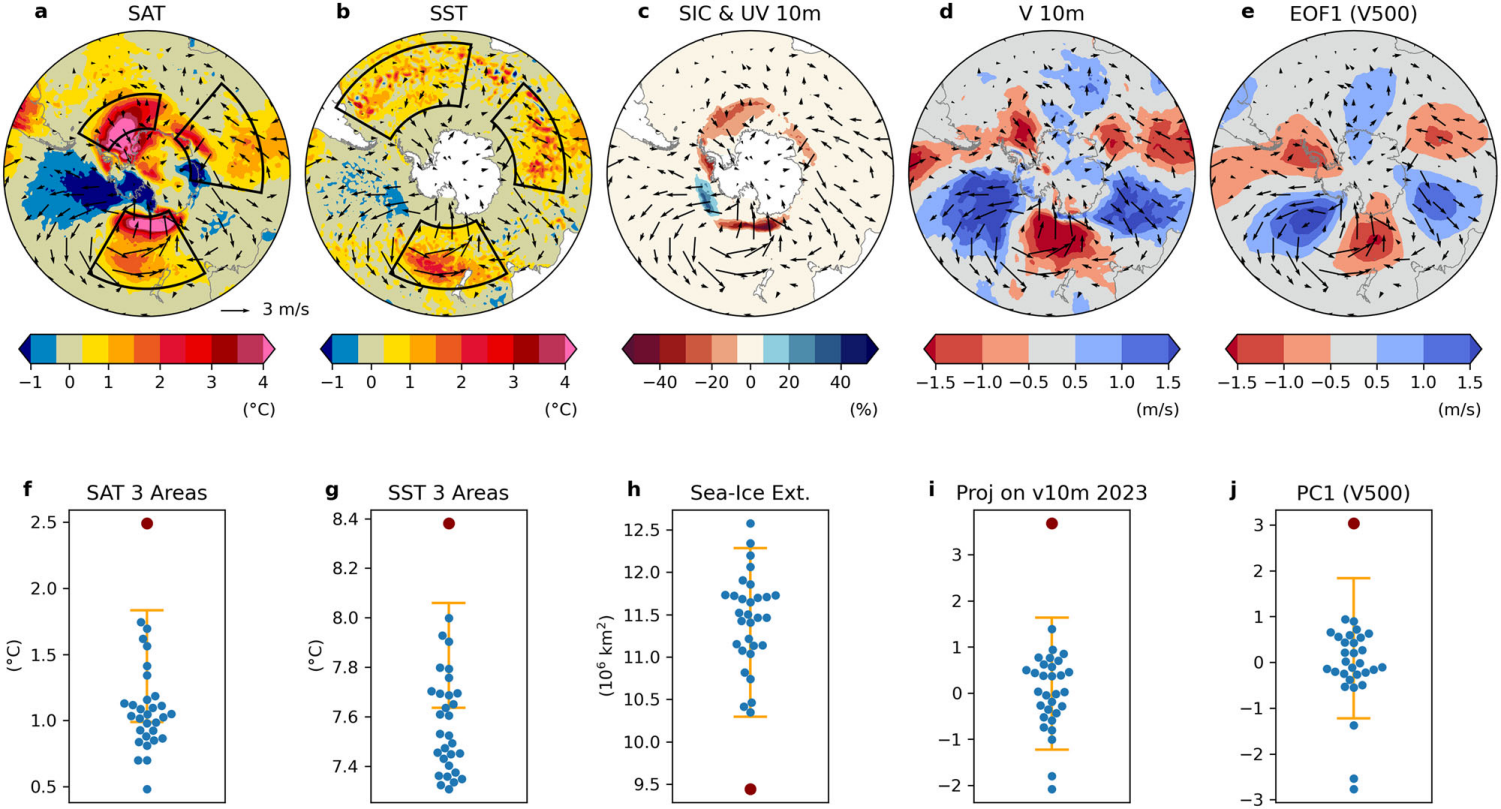
(Top panels) Southern Hemisphere anomalies from March to August 2023 relative to the 1993–2022 climatology. Color shading for **a** Surface Air Temperature (SAT), **b** Sea Surface Temperature (SST), **c** Sea-Ice Concentration (SIC), **d** 10-m Meridional Wind Speeds (V10m), and **e** the first Empirical Orthogonal Function (EOF1) of 500 hPa meridional winds with 10-m wind speed anomalies represented as vectors. The bottom panels display the corresponding seasonally (March–August) averaged data for each year, with the red dot indicating the 2023 value, blue dots indicating 1993–2022 values, and the yellow bar representing the 5th–95th percentile range of the 2023 value estimation based on a linear regression model for the period 1993–2022. **f**, **g** show area-averaged SAT and SST, respectively. **h** presents sea-ice extent, **i** shows projection coefficients of 10-m meridional wind speeds onto its 2023 pattern as shown in (**d**), and **j** displays the time coefficients of EOF1 as shown in (**d**).

In contrast to the subtropical Northeastern Atlantic, the warming pattern in the Southern Ocean, which exhibited abnormal record-breaking conditions from February through September, does not have a direct connection to local TOA net radiation but was closely related to abnormal atmospheric circulation anomalies. Figure 6a–d, f–i indicates that the anomalously warm SST and SAT and reduced sea-ice averaged between March and August 2023 were closely associated with a wave number 3 pattern in northerly wind anomalies in the Pacific, Atlantic and Indian sectors of the Southern Ocean. This pattern is known to play an important role in Southern Ocean climate including influencing Antarctic sea ice^56–58^.

To better understand the wave number 3 pattern in 2023, an Empirical Orthogonal Function (EOF) analysis was conducted on the 500 hPa meridional wind, averaged from March to August for each year, south of 20°S (Fig. 6e). It is found that the amplitude of the wave number 3 pattern was exceptionally high between March and August in 2023, as shown by +3 standard deviation of the principal component, far exceeding the previous highest value of +1 standard deviation, and well above an abnormal record-breaking condition (Fig. 6j). Abnormal record-breaking conditions occurred in all time series of the separate MAM and JJA seasons of 2023, as well (not shown). Moreover, the wave number 3 pattern is evident in AE development in 2023 (Supplementary Fig. 2), indicating that this pattern plays an important role in shaping heat distribution in the atmosphere. Previous studies of exceptionally low sea ice in 2023^23,28^ discussed the potential influence of atmospheric circulation anomalies on the low sea ice conditions, but the role of the wave number 3 pattern was not identified. The wave number 3 pattern is evident even if we apply the AB-test at each grid point of geopotential height at 500 hPa (Supplementary Fig. 8). Elsewhere over the globe, such prominent anomalies in atmospheric circulation are not found.

What might have caused the exceptional wave number 3 pattern in southern hemisphere atmospheric circulation? This pattern is known to be a leading feature of interannual variability in the region. A previous study^57^ suggested that changes in tropical deep convection, whether due to natural variability or climate change, exert a strong influence on this pattern. However, it should be noted that the wave number 3 pattern itself may not fully explain the exceptional condition of the Southern Ocean especially the overall decline of the sea-ice in 2023 (Fig. 1f). Further research is needed to fully understand the exceptional conditions in the Southern Ocean, including the role of tropical convection anomalies and the underlying causes of the overall sea-ice decline.

The extratropical Northwestern Pacific is unusual in that abnormal record-breaking conditions also occurred for several months in 2022 (Fig. 2a). The persistent warmth in this region is likely related to the ocean’s response to an anomalously weak Aleutian low on decadal timescale, which further weakened during the winters between 2021 and 2023, associated with the 3-year La Niña^59,60^. Weak Aleutian lows in multiple years cause warm anomalies in this region in association with a negative Pacific Decadal Oscillation^61,62^. However, the transition to El Niño in 2023 has not resulted in an anticipated intensification of the Aleutian Low through atmospheric teleconnections^63^, and therefore temperature anomalies in this region remain very high.

## Discussion

While the observations used in this study capture the main features of the exchange of heat between space, the atmosphere, and the oceans during the anomalous 2023–2024 period, open questions remain. There has been recent progress in identifying some of the causes of the positive trend in EEI^1,18,19,33,45,64^ but there are still large uncertainties, concerning for example the roles of anthropogenic aerosols and of changes in clouds. As in situ observations in mid- and deep-ocean layers are sparse, there is also some uncertainty related to heating of these layers. Further questions concern the role of ocean heat transports, for example, the extratropical Northwestern Pacific (Fig. 1), where changes in the Kuroshio Current likely played a role^59,60^.

Our results show that the 2023–2024 extremes cannot be explained as simple extensions of long-term anthropogenic trends; instead, there was a critical role for regional processes, some of which are linked to interannual modes of variability (ENSO, wavenumber-3, Aleutian Low) which acted to amplify warming. There is a need for more detailed process and attribution studies to elucidate the causes and effects, including timing, of the exceptional warming in each of the regions we have highlighted. For example, why did exceptional warming appear first in the Southern Ocean (Fig. 2)? Will the exceptional wavenumber 3 pattern in atmospheric circulation recur in future years? Another aspect that merits further investigation concerns the changes in the tropical North Atlantic. Our analysis for this region showed a close link between cloud cover, TOA radiation and record-breaking SST anomalies, suggesting a potential positive feedback between reductions in low cloud and warmer SSTs in this region^65^. How important was this feedback and might it recur in future years? An additional important question is whether the warming patterns observed in the different ocean basins in 2023 were causally connected, e.g. through changes in the atmospheric circulation; the answer to this question certainly influences how these patterns will further evolve.

Since the peak of the heat extremes in late 2023 and early 2024, SST anomalies have dropped in many regions (Fig. 2 and Supplementary Fig. 9), except for the Northwest Pacific where SST anomalies have further increased well above 2 °C, where ocean circulation anomalies play important role in SSTs^59,60^. Subseasonal-seasonal forecast systems predict (Supplementary Fig. 9) that SSTs in this region will stay well above climatological values for the next 6 months and will extend further east towards the west coast of North America, associated with a weak Aleutian Low. SST anomalies in the central tropical Pacific are predicted to turn negative, indicating La Niña-like conditions, with associated positive SST anomalies in the western tropical Pacific; these are forecasted to persist for the next 6 months. For the North Atlantic, forecast systems predict moderately warm SSTs in the subtropical Northeastern Atlantic, with higher anomalies farther north. On the other hand, SSTs over the Southern Ocean are predicted to increase again in response to a reoccurring wavenumber 3 pattern.

A vital question is whether the exceptional events of 2023–2024 have implications beyond 2024—for expected climate change in the years and decades ahead. A basic but crucial point is that the positive trend in EEI since 2000 means that global warming (measured by heat uptake by the Earth’s climate system) is accelerating. What is not yet clear is if the drop in EEI in 2024 is merely a brief respite from the multi-decadal upward trend, and to what extent this acceleration in heat uptake will influence trends in surface temperature over the years and the decade to come. On decadal timescales we expect a positive correlation between changes in EEI and changes in surface temperature; however, there is variability in this relationship associated with vertical redistribution of heat within the ocean^66^, which can temporarily enhance or offset the EEI influence. Nevertheless, in the presence of a positive trend in EEI, natural fluctuations that perturb the global energy budget—such as those associated with ENSO cycles—will sooner or later have larger and sometimes record-breaking impacts, including on surface temperatures, because the associated EEI anomalies will be larger than they were in the past. The 2023–2024 period is a clear example of this, and similar events can be expected in future.

## Methods

### Datasets

The datasets analyzed in this study are listed in the Table 1.

**Table 1:** Datasets analyzed in this study

Data set name	Variables	Resolution	Period analyzed in this study	Reference No.
OI-SST, version 2.1	SST	0.25° × 0.25°, daily	January 1993–December 2024	^72^
ERA5	Three-dimensional temperature, geopotential, specific humidity, and wind speeds, and surface air-temperature, latent heat flux, sea-ice concentration	0.25° × 0.25°, monthly	January 1993– December 2024	^73^
ORAS5	Potential temperature, salinity, mixed-layer depth	Curvilinear 1021 × 1442 grids, monthly	January 1993– December 2024	^68^
EN 4.2 Ocean Analysis	Potential temperature and salinity	1° × 1°, monthly	January 1993– November 2024	^69^
IAP Ocean Heat Content Analysis, version 4	0–100 m and 0–300 m OHC	1° × 1°, monthly	January 1993– December 2024	^71^
JMA Ocean Analysis, version 7.3.1	Temperature and salinity	1° × 1°, monthly	January 1993– December 2023	^70^
CERES-EBAF TOA, version 4.2	Net, shortwave, and longwave radiation at TOA	1° × 1°, monthly	March 2000– July 2024	^74^
CERES-EBAF, version 4.2	Cloud fraction, and shortwave radiation at the surface	1° × 1°, monthly	March 2000– May 2024	^74^
NOAA Multivariate ENSO Index, version 2	Index for El Nino and La Nina	Average of consecutive 2 months	January 1993–December 2024	^75^
NOAA Sea-Ice Index, Version 3	Sea-ice extent	Monthly	January 1993–December 2024	^76^

### Abnormal record-Breaking test

We have introduced a simple statistical analysis, the “Abnormal record-Breaking (AB) test”—a time series analysis which examines whether a specific observations satisfies two conditions: (1) it is record-breaking, i.e., it is has an unprecedentedly high (e.g., for temperatures) or low (e.g., for sea-ice) value, and (2) it is an outlier of the expected range estimated from the past trend, surpassing the threshold for the top 5%, thus deemed significant at the 5% level in a one-sided test.

The expected range is estimated by a linear regression analysis using data leading up to the year of interest. As global warming has accelerated in recent decades^4,7^, it is appropriate to estimate the trend using recent data. It is important to note that we use the 30-year period of 1993–2022 for the trend estimation. (Shorter time periods are used for selecting variables with datasets that begin after 1993.) The trend calculation period of 30 years is used to account for potential problems of too long and too short calculation period. If the trend is calculated over a much longer period (e.g., 100 years), due to the warming rate is stronger in recent years than 100 years ago, the recent temperature data will be judged as abnormal, simply because of the contrast between the recent warming rate and that of a century ago. On the other hand, if the trend is calculated over too short a time period, the uncertainty in the estimate may be too large. We believe that 30 years is an appropriate period to balance these two effects. The concept of the AB test is further explained in supplementary material using global air temperature as an example time series (Supplementary Fig. 1).

### Atmospheric energy

Atmospheric energy (AE) is calculated from ERA5 monthly air-temperature, specific humidity, geopotential, and surface pressure. We follow the formulation described in ref. ^6^. Their equation of AE per unit area on height coordinate,

1 $${E}_{A}={\int }_{{Z}_{s}}^{{Z}_{{TOA}}}\rho \left({c}_{V}T+{gz}+{L}_{e}q+\frac{{V}^{2}}{2}{dz}\right)$$

where ${E}_{A}$ is the AE, $z$ is the height, ${Z}_{s}$ is the surface height, ${Z}_{{TOA}}$ is the height of the top of the atmosphere, $T$ is the temperature, $\rho$ is the density of the air, $q$ is the specific humidity, $V$ is the wind speed, $g$ is the gravity acceleration, ${c}_{V}$ is the specific heat at constant volume, ${L}_{e}$ is the latent heat for condensation and evaporation for the temperature above 0 °C or the latent heat for sublimation for the temperature below it. Geopotential energy was referenced to the surface in ref. ^6^, whereas in our equation, it is referenced to geopotential zero. This difference is negligible for the anomaly results presented in this paper. In order to calculate the AE using monthly data on pressure coordinate, Eq. (1) is converted to pressure coordinate with ignoring the velocity term (kinetic energy) as:

2 $${E}_{A}={\int }_{0}^{{p}_{s}}\quad \left({c}_{V}\frac{{\rm{T}}}{g}\quad +z\quad +\quad {L}_{e}\quad \frac{q}{g}\right){dp}$$

We ignored kinetic energy in our calculation, because anomalies of kinetic energy over the globe is negligibly small^6^ for our study, where magnitudes of OHC and TOA radiation is one order larger than the AE. We also examined AE calculated based on ref. ^49^, which is one of different formulations of AE^49,67^, and found that the Fig. 1a calculated by both methods produces indistinguishable curves.

The global AE time series analyzed by ref. ^6^ available at https://www.wdc-climate.de/. The RMSE between their AE and ours is 0.2 ZJ, consistent with their uncertainty among different source data. Therefore, we estimate the uncertainty in AE to be 0.2 ZJ.

### Ocean heat content

We calculate the Ocean Heat Content (OHC) from spatially three-dimensional potential temperatures (ORAS5^68^, EN4.2^69^) or from in-situ temperatures provided by Japan Meteorological Agency (JMA)^70^ (version 3.7.1) and respective salinity data using TEOS-10 gsw python toolkit (https://teos-10.github.io/GSW-Python/). We also use OHC data for 0–100 and 0–300 m layer thickness provided by Institute of Atmospheric Physics (IAP) (version 4) in China^71^. We thus have four OHC estimates based on ocean temperature and salinity. In addition, the vertically integrated OHC over the entire ocean water column was estimated from satellite altimetry and space gravimetry^3^.

Ref. ^3^ directly provides the total ocean heat uptake (OHU), the time derivative of OHC, from the ocean surface to the bottom of ocean over the period 2002–2021. The top 300 m OHU is estimated using the four OHC data products. In Fig. 4a, b we compute the ocean heat uptake below 300 m depth by taking the difference between ref. ^3^ estimate of the total OHU and the 0 to 300 m OHU computed from the four OHC products. The uncertainty for the entire ocean water column is derived from ref. ^3^ OHU uncertainty estimate, and the 0–300 m OHU uncertainty is given by the standard deviation of the OHU estimates from the four OHC datasets. Considering both are independent, the OHU uncertainty below 300 m depth is estimated. Note that over the periods of interest (i.e. 2009–2010 for Fig. 4a and 2015–2016), the difference between TOA net radiation budget minus the AE derived from ERA5 and ref. ^3^ total OHU is less than 1 ZJ meaning that the global energy budget is closed with these datasets within the error bars.

For the global energy budget over the period 2022–2023, the total OHU is not available from ref. ^3^ dataset because satellite altimetry data is not available yet over the second half of 2023. Therefore, we adopt a different approach to estimate the OHU below 300 m depth in Fig. 4c. Given the precise closure of the energy budget over 2009–2010 and 2015–2016, we assume the energy budget is also closed in 2022–2023 and we infer the ocean heat uptake below 300 m depth by taking the difference between the TOA net radiation budget minus the AE derived from ERA5 and the 0 to 300 m OHU computed from the four OHC products. We apply the same uncertainty to the 2022–2023 OHU below 300 m depth as the uncertainty in the 2015–2016 OHU below 300 m depth.

### Mean-retained anomaly

In climate science research, the amplitude of seasonal variations often exceeds the magnitude of the climate variability or change being studied. To isolate climate variability or change, it is common to use anomalies, which represent the difference between observed values and climatology. The conventional anomaly for a monthly time series can be expressed as:

3 $$u^{\prime} ({mo},{yr})=u({mo},{yr})-\bar{u}({mo})$$

where *u* is the dependent variable being analyzed, *mo* is the calendar month, and *yr* is the year. Prime (‘) indicates the anomaly, and overbar indicate the climatology:

4 $$\bar{u}(mo)=\frac{1}{Yr}\sum _{yr}u(mo,yr)$$

where ${Yr}$ is the number of years used to calculate the climatology.

However, for certain variables, it is important to know how the time-averaged value for a given period relates to zero. One such variable is the global TOA net radiation. Positive and negative time-averaged values of global TOA net radiation indicate whether the Earth is absorbing or releasing heat, respectively. This information is not directly discernible from the time mean of conventional anomalies. To address this in some cases, a 12-month running mean of observed value is shown (e.g., Fig. 21 of ref. ^21^). The drawback using a 12-month running means is that it becomes difficult to know the contribution of individual months.

To avoid the limitations of both conventional anomalies and 12-month running means, we propose a mean-retained anomaly, defined as:

5 $${u}^{\ast }(mo,yr)=u^{\prime} (mo,yr)+\langle u\rangle$$

where asterisk (*) indicates the mean-retained anomaly, and bracket indicates the simple time mean as:

6 $$\langle u\rangle =\frac{1}{12}\frac{1}{Yr}\sum _{yr}\sum _{mo=1}^{12}u(mo,yr)=\frac{1}{12}\sum _{mo=1}^{12}\bar{u}(mo),$$

where 12 is number of calendar months. The time average of mean-retained anomalies over single or multiple years is identical to the corresponding time average of raw values. This can be demonstrated using the sum of 12 months for a given year:

7 $$\begin{array}{c}\begin{array}{c}\begin{array}{c}\displaystyle \sum _{mo=1}^{12}u\ast (mo,yr)=\displaystyle \sum _{mo=1}^{12}\{u{\rm{^{\prime} }}(mo,yr)+\langle u\rangle \}\\ \,\,\,\,=\displaystyle \sum _{mo=1}^{12}\{u(mo,yr)-\bar{u}(mo)\}+12\langle u\rangle \\ \,\,\,\,=\displaystyle \sum _{mo=1}^{12}u(mo,yr)-12\langle u\rangle +12\langle u\rangle \\ \,\,\,\,=\displaystyle \sum _{mo=1}^{12}u(mo,yr).\end{array}\end{array}\end{array}$$

In this example, the sum is taken for 12 months of a calendar year for simplicity, but the identity of the 12-month sum of the mean-retained anomaly with the original data holds any sequential 12 months (e.g., from July to next year June), as the second and third terms in the right-hand side cancel each other out. Similarly, the identity holds for the sum or average of consecutive months whose length is a multiple of 12 months.

The mean-retained anomaly is particularly useful for variables for which zero is important and the observed value is close to zero, as global TOA net radiation. For this reason, we apply the mean-retained anomaly to this variable. Conversely, for variables with values far from zero, such as global shortwave radiation and longwave radiation, the mean-retained anomaly offers no advantage over the conventional anomaly. The mean-retained anomaly may also be useful for other variables in climate science beyond those examined in this paper. In particular, it could be valuable for variables like precipitation in arid regions, where values near zero are significant.

### Definitions of areas

The areas shown in Fig. 1h are as follows: for the extratropical Northwestern Pacific a box over 35**°**–45**°**N, 130**°**E–170**°**W, for the subtropical Northeastern Atlantic a box over 10**°**–30**°**N, 40**°**–15**°**W, for the tropical Pacific a polygon of (15**°**S, 150**°**E), (15**°**S, 75**°**W), (10**°**N, 75**°**W), (10**°**N, 80**°**W), (15**°**N, 80**°**W), (15**°**N, 15**°**0E), for the Southern Ocean three boxes over 60**°**–40**°**S, 40**°**–100**°**E, 65**°**–45**°**S, 150**°**E–150**°**W, and 60**°**–35**°**S, 60**°**W–10**°**E.

### Copernicus Climate Change Service (C3S) subseasonal to seasonal forecasts

Freely available monthly mean ensemble anomalies from 8 modeling centers (ECMWF, NCEP, DWD, CMCC, METEO-France, JMA, ECCO and UKMO) were used to compile the multi-model mean SST and sea level pressure anomalies in Supplementary Fig. 10. These monthly updated forecasted products for SST and other physical variables have a horizontal resolution of nominal 1 degree and allow forecasts of up to 6 months ahead of time. Area-averaged anomalies are provided for some selected regions, following the area definitions above.

## Supplementary information

Supplementary Material

## Data Availability

Data are available from the following sites: OI-SST, https://psl.noaa.gov/data/gridded/data.noaa.oisst.v2.html; ERA5, https://www.ecmwf.int/en/forecasts/dataset/ecmwf-reanalysis-v5; SIE, https://nsidc.org/arcticseaicenews/sea-ice-tools/; ORAS5, https://www.ecmwf.int/en/elibrary/80763-ocean5-ecmwf-ocean-reanalysis-system-and-its-real-time-analysis-component; Grid-cell information of ORAS5, https://icdc.cen.uni-hamburg.de/thredds/catalog/ftpthredds/EASYInit/oras5/ORCA025/mesh/catalog.html; CERES-EBAF, https://asdc.larc.nasa.gov/data/CERES/EBAF/TOA_Edition4.2/; Multivariate ENSO index, https://psl.noaa.gov/enso/mei/; Total ocean heat content from satellite altimetry, https://www.aviso.altimetry.fr/en/data/products/ocean-indicators-products/ocean-heat-content-and-earth-energy-imbalance/global-ocean-heat-content-change-and-earth-energy-imbalance.html; Copernicus Climate Change Service (C3S) seasonal forecast data, https://cds.climate.copernicus.eu/cdsapp#!/dataset/seasonal-postprocessed-single-levels?tab=overview.

## Code availability

All code is available upon reasonable request to the corresponding author. Sample Python code for the AB-test is publicly available at https://github.com/sminobe/AB-test.
