# Supplementary material for: Global and regional drivers for exceptional climate extremes in 2023-2024: beyond the new normal
Source: NPJ Clim Atmos Sci. 2025 Apr 7;8(1):138. doi: 10.1038/s41612-025-00996-z (PMC11972963; doi:10.1038/s41612-025-00996-z)
Supplement: Supplementary file 1 — Supplementary Material [file 41612_2025_996_MOESM1_ESM.pdf]

Supplementary Materials

Supplementary Table 1. Datasets used in Supplementary figures in addition to the datasets listed in Table 1.

Data set name	Variables	Resolution	Period analyzed in this study	Reference No.
ERSST, version 5	SST	2.0°×2.0°, monthly	January 1950-September 2024	77
MODIS Cloud Properties Product (MCD06)	Total cloud ratio and low cloud ratio	1.0°×1.0°, monthly	July 2002 – September 2024	78
MODIS standard atmosphere product (MOD08 & MYD08)	Aerosol optical thickness	1.0°×1.0°, monthly	July 2002 – October 2024	79,80

Supplementary Table 2. Heat content changes in the global atmosphere and ocean and integrated TOA net radiation over the globe (in ZJ), with contribution ratio in the tropics (30S-30N) indicated in the parentheses. For the atmospheric energy (AE), the value is estimated as the RMES of AE between the present study and that used by von Schuckman et al. (2023). Their AE data is provided though World Data Center for Climate as globally integrated, annually sampled, and ENSO removed AE. For the ocean heat content (OHC) values in the 0-100 m and 100-300 m depth ranges, the values listed after the '±' represent one standard deviation as estimated from four ocean reanalysis and analysis products. For TOA net radiation integrated over the globe for a one-year period, its standard deviation is based on monthly error estimates between Terra and Aqua observations. The OHC below 300 m is estimated by the difference between the time integration of TOA net radiation and 0-300 m OHC change. The value shown after the '±' for OHC below 300 m includes the uncertainty associated with discrepancy between the TOA net radiation and OHC change estimated from satellite observation of sea-surface height and gravity.

	2010 Feb - 2009 Feb	2015 Dec - 2014 Dec	2023 Nov - 2022 Nov
AE Change	2.8±0.2 (82%)	1.9±0.2 (126%)	4.7±0.2 (72%)
0-100 m OHC Change	27.6±1.0 (107%)	29.5±2.0 (84%)	43.7±1.1 (95%)
100-300 m OHC Change	-24.5±4.4 (86%)	-21.6±1.4 (115%)	-29.4±2.5 (101%)
OHC Change below 300 m	4.4±7.8	6.9±5.7	10.7±5.7
Time integration of TOA Net Radiation	10.3±1.4	16.6±1.4	29.6±1.4

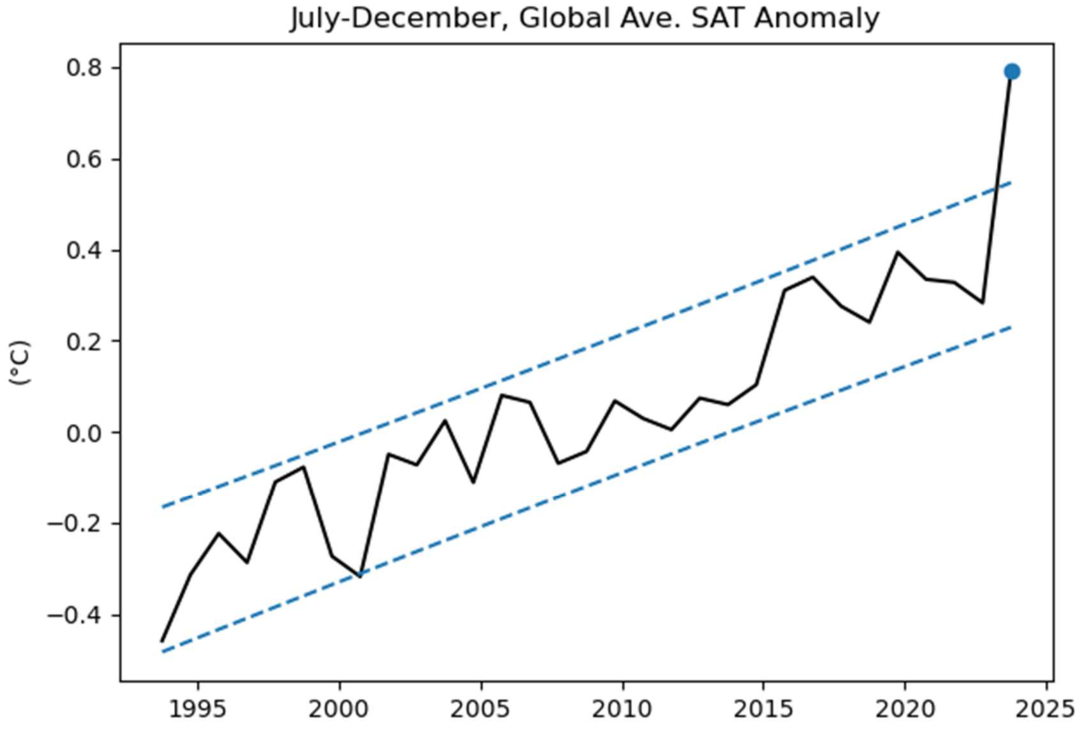

Supplementary Fig. 1. Illustration of the AB-test applied to global Surface Air Temperature (SAT) anomalies for July-December. Dashed lines delineate the expected data range at 90% confidence, based on the linear trend from 1993 to 2022. Values surpassing the upper limit of this range are within the top 5%, thus deemed significant at the 5% level in a one-sided test. The linear trend is calculated using data up to one year prior to the year being tested (2022 for testing 2023 data in this instance). When we test the data in 2022, the trend is estimated using the data for 1993-2021.

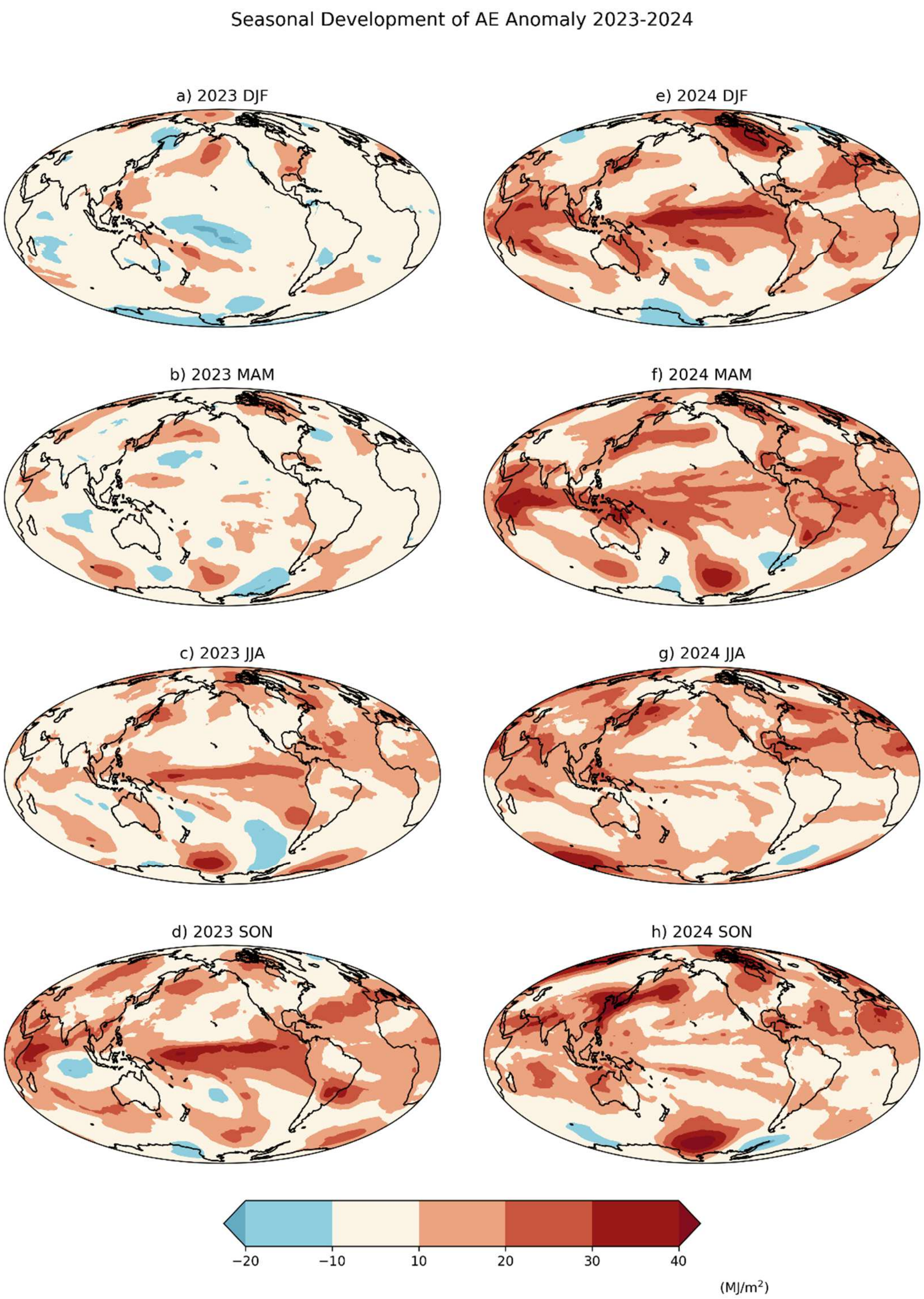

Supplementary Fig. 2: AE anomaly evolution from 2023 DJF to 2024 SON.

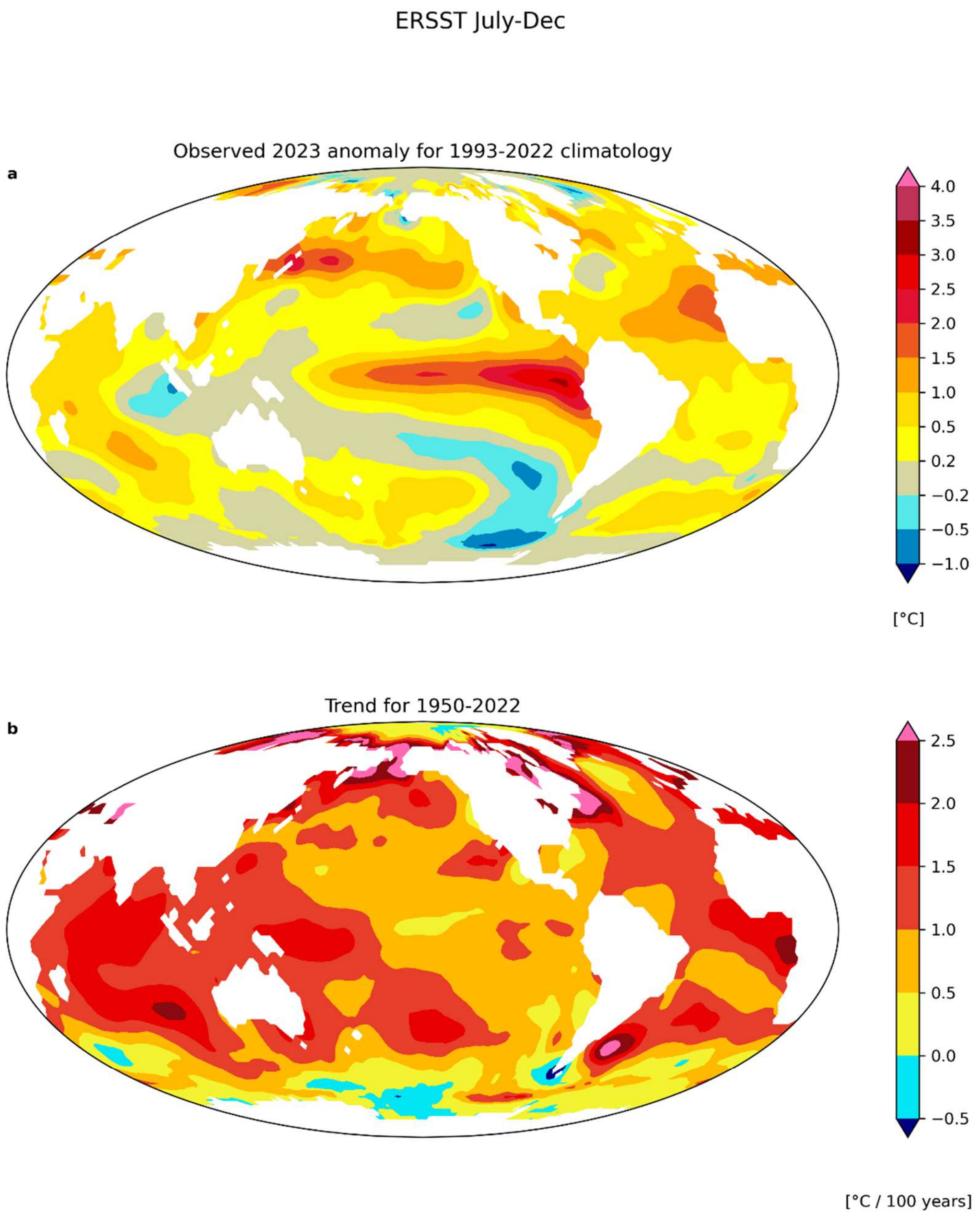

Supplementary Fig. 3. Comparison of 2023 anomaly and trend for 1950-2022 period for July-December season based on Extended and Reconstructed SST version 5[72].

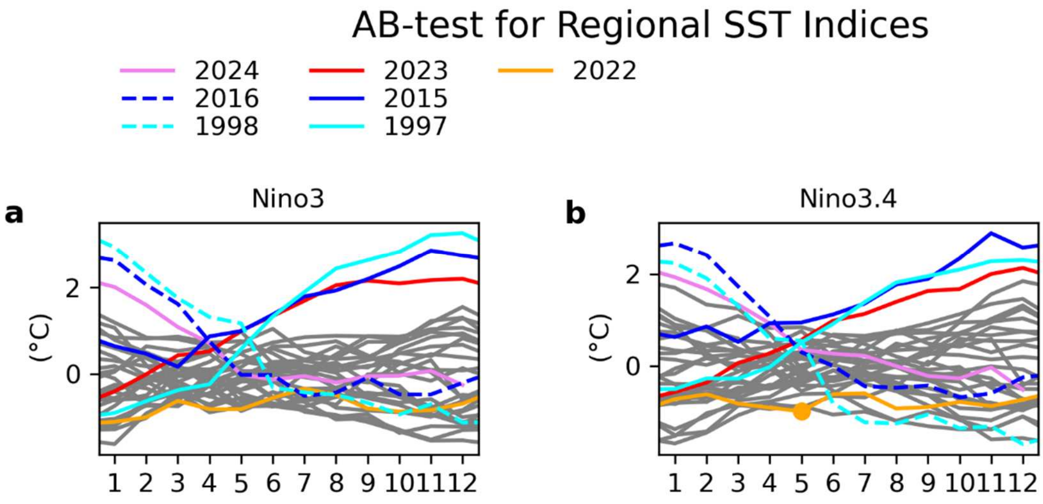

Supplementary Fig. 4. AB-test for (a) Nino3 and (b) Nino3.4 indices. These indices are calculated from OI-SST.

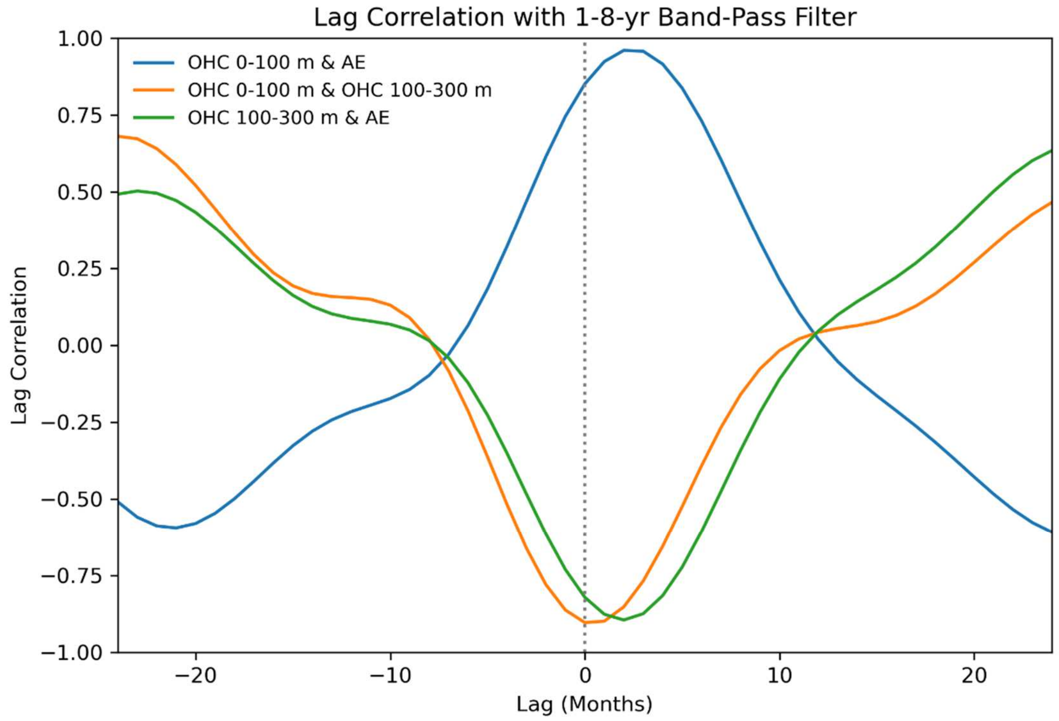

Supplementary Fig. 5. Lag correlation between near-surface (0-100 m) OHC, subsurface (100-300 m) OHC, and AE with a band-pass filter, which extracts an interannual (one-to-eight year period) component. Positive lags correspond to the first variable shown in legend leading the second variable.

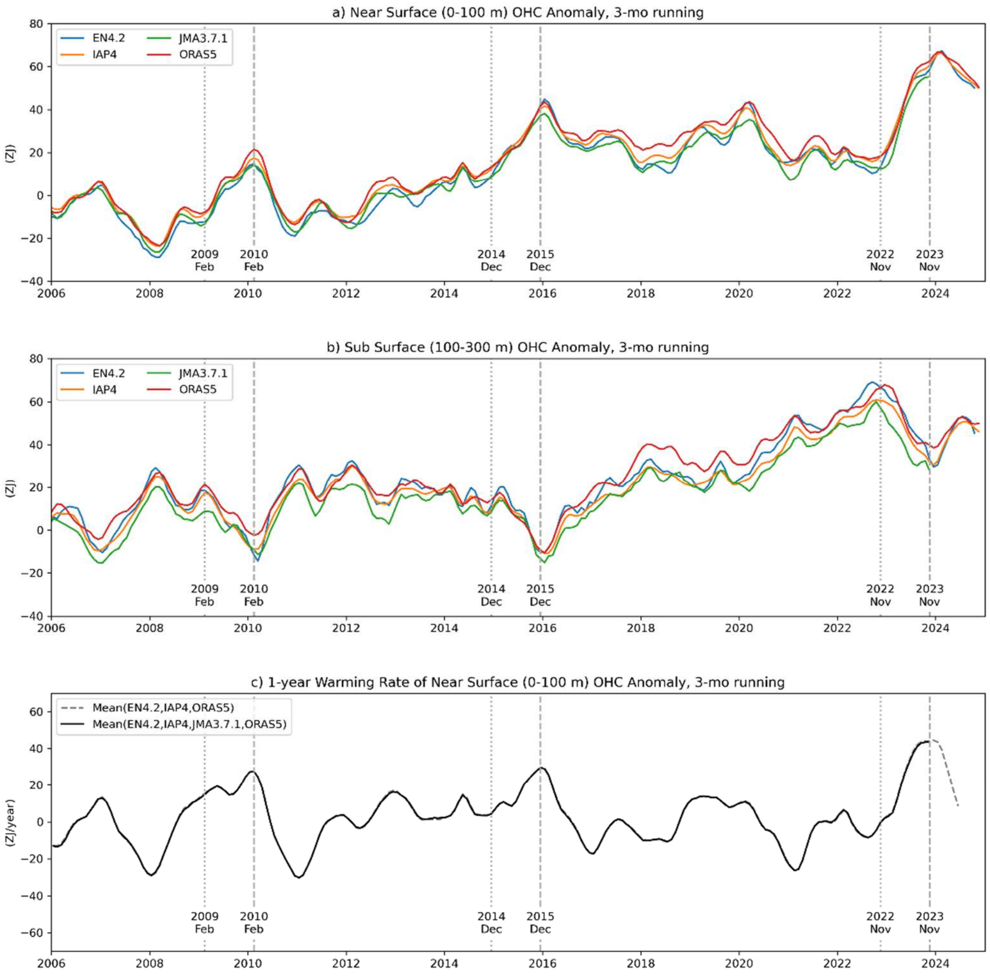

Supplementary Fig. 6. (a) 0-100 m and (b) 100-300 m OHC of four products, and (c) 1-year warming rate of the 0-100 m OHC. The vertical dotted and dashed lines in each panel indicate the one-year period during which the heat budget analysis of Fig. 4 is conducted.

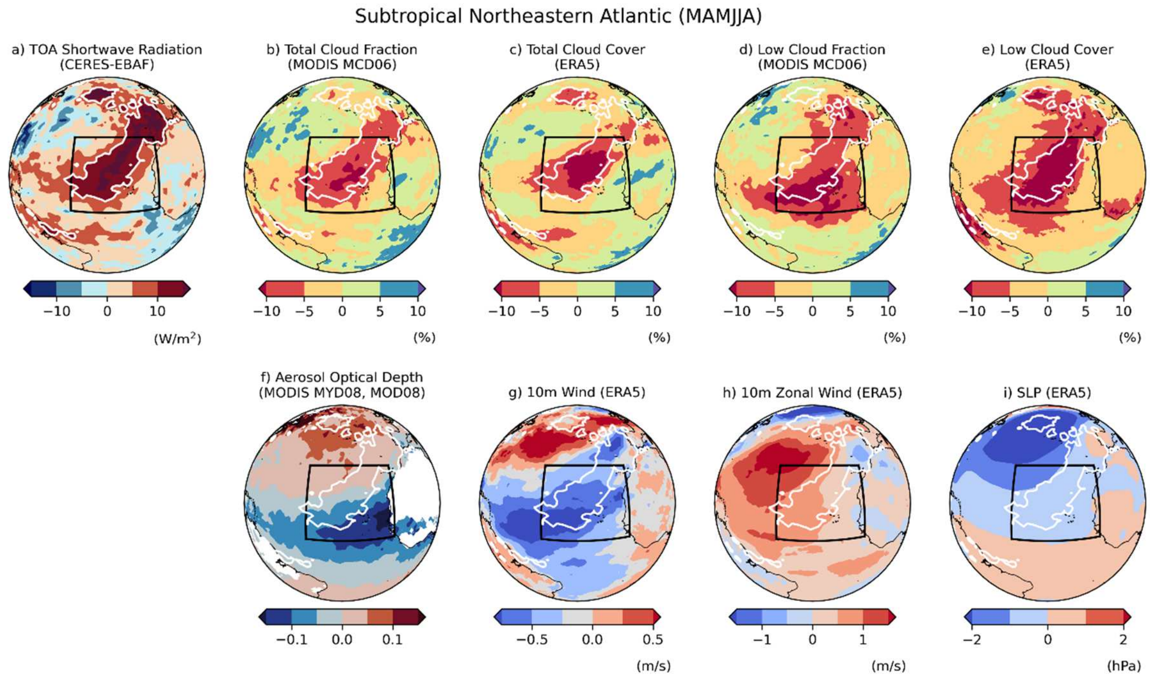

Supplementary Fig. 7: Anomalies of (a) TOA shortwave radiation, (b) total cloud fraction observed by MODIS, (c) total cloud cover of ERA5, (d) low cloud fraction observed by MODIS, (e) low cloud cover of ERA5, (f) aerosol optical depth observed by MODIS, (g) 10-m scalar wind speed of ERA5, (h) 10-m zonal wind speed of ERA5, and (i) SLP of ERA5 in 2023 MAMJJA. The white contours are 10 W/m^2 of TOA shortwave radiation in panel (a). Panel (a) is also shown in Fig. 5, but are reproduced here for the convenience of readers. The cloud data of MODIS are taken from MCD06 dataset and aerosol optical depth is taken from MYD08 and MOD08 datasets. Anomalies are calculated relative to the climatologies for the periods 1993–2022, 2000–2022, and 2003–2022 for ERA5, CERES, and MODIS data, respectively, reflecting the limited availability of the latter two datasets. The results remain essentially unchanged if a uniform reference period of 2003–2022 is used for all datasets (not shown).

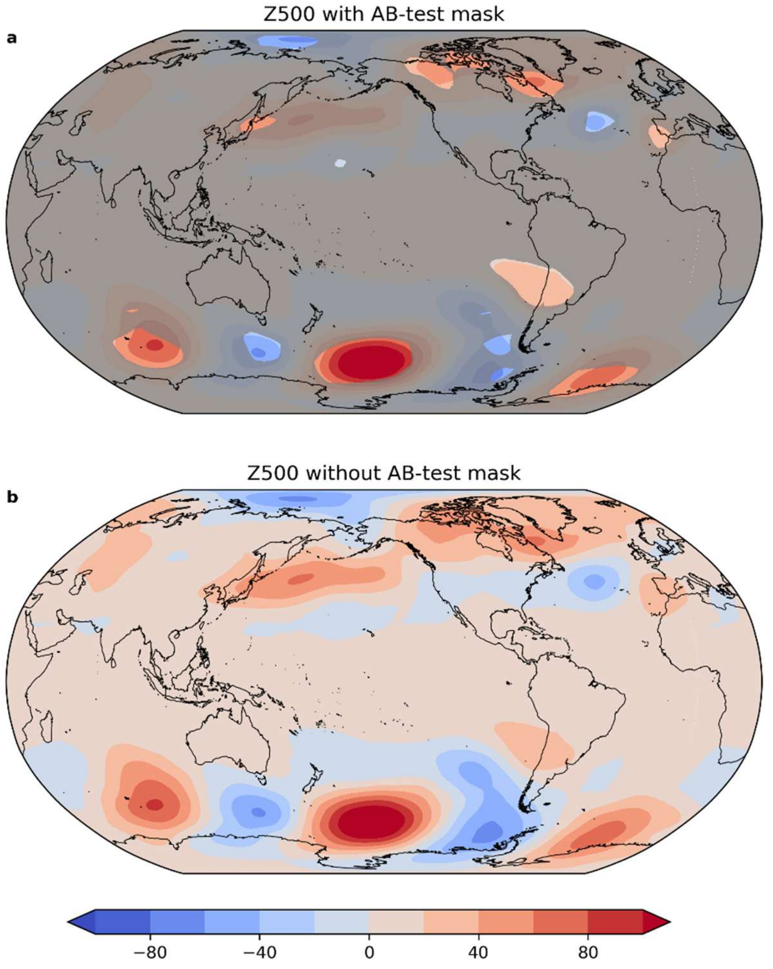

Supplementary Fig. 8: 500 hPa (left) geopotential height anomalies from March to August in 2023 with (top) and without (bottom) the AB-test masks. Anomalies are relative to the 1993-2022 climatology. In the top panel, the values that do not pass the AB-test are shown with the grey overlapping shading. The wave number three pattern around Antarctica is evident. This figure also suggests the propagation from the high latitude southeastern South Pacific to lower latitude toward South America, and propagation from the Labrador Sea, crossing the North Atlantic to Spain with a hint of North Atlantic Oscillation.

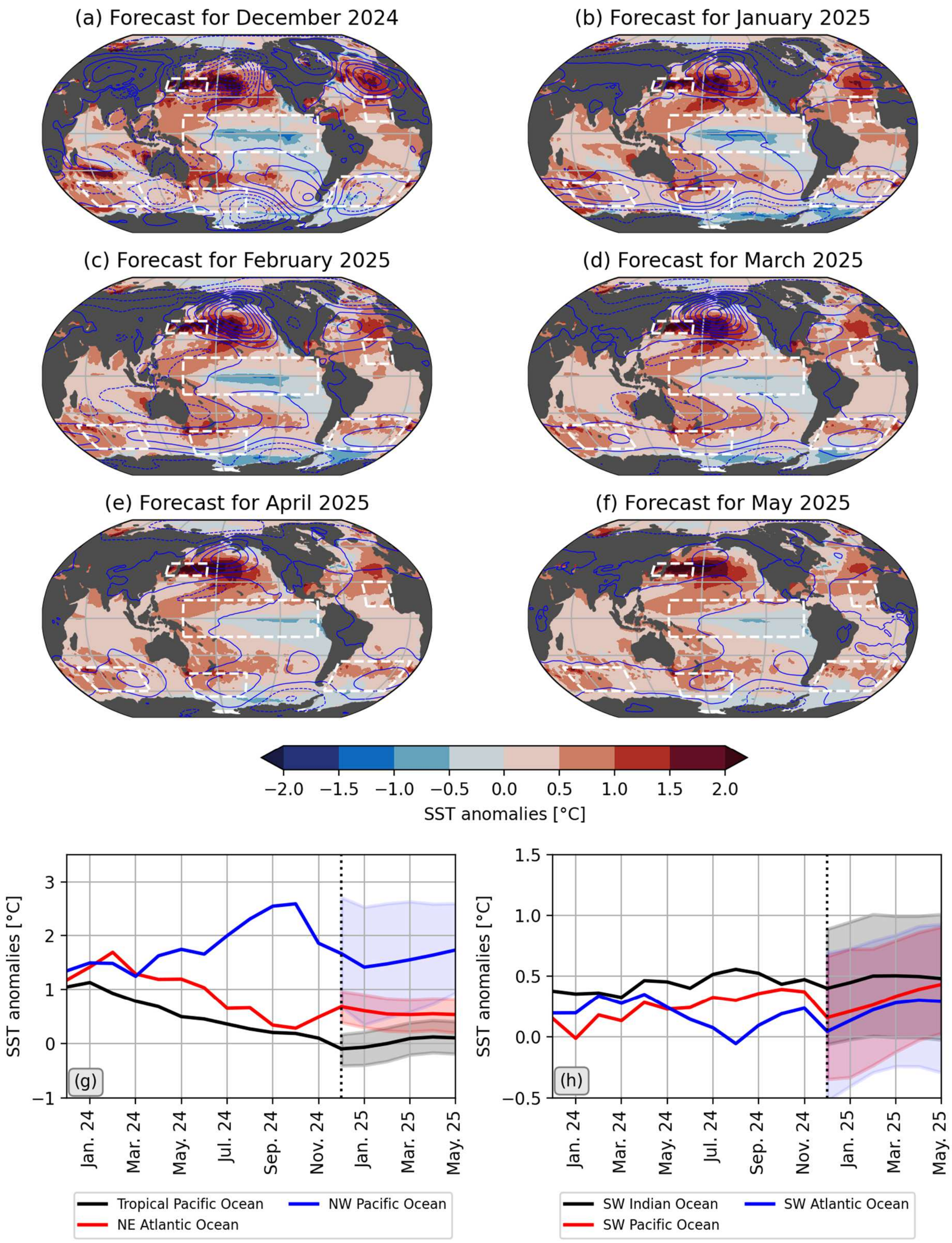

Supplementary Fig. 9: (a-f) C3S multi-model mean forecasted SST anomalies for the upcoming 6 months (December 2024 to May 2025). Blue contours represent multi-model mean sea-level pressure anomalies, with a contour interval off 1 hPa (negative anomalies are represented by dashed contours). (g, h) Area-averaged anomalies for selected regions (white boxes in (a-f)) are shown by color-coded solid lines. Reanalysis period is to the left of vertical

dashed line while the forecast period to right where the shading represents the multi-model spread.

References for Supplementary Materials

77. Huang, B. et al. NOAA Extended Reconstructed Sea Surface Temperature (ERSST), Version 5. NOAA National Centers for Environmental Information https://doi.org/10.7289/V5T72FNM (2017).
78. Platnick, S. et al. The MODIS Cloud Optical and Microphysical Products: Collection 6 Updates and Examples From Terra and Aqua. IEEE Trans. Geosci. Remote Sensing 55, 502–525 (2017).
79. Platnick, S., Hubanks, P., Meyer, K. & King, M. D. MYD08_M3 MODIS/Aqua Aerosol Cloud Water Vapor Ozone Monthly L3 Global 1Deg CMG. NASA Level 1 and Atmosphere Archive and Distribution System Distributed Active Archive Center https://doi.org/10.5067/MODIS/MYD08_M3.006 (2015).
80. Platnick, S., Hubanks, P., Meyer, K. & King, M. D. MOD08_M3 MODIS/Terra Aerosol Cloud Water Vapor Ozone Monthly L3 Global 1Deg CMG. NASA Level 1 and Atmosphere Archive and Distribution System Distributed Active Archive Center https://doi.org/10.5067/MODIS/MOD08_M3.006 (2015).

Data Availability

Extended and Reconstructed SST:
https://psl.noaa.gov/data/gridded/data.noaa.ersst.v5.html
MODIS MCD06 data:
https://ladsweb.modaps.eosdis.nasa.gov/archive/allData/62/MCD06COSP_M3_MODIS
MODIS MOD08 data:
https://ladsweb.modaps.eosdis.nasa.gov/archive/allData/61/MOD08_M3/
MODIS MYD08 data:
https://ladsweb.modaps.eosdis.nasa.gov/archive/allData/61/MYD08_M3/
